# Reciprocity, Fairness and the Financial Burden of Undertaking COVID-19 Hotel Quarantine in Australia

**DOI:** 10.1093/phe/phad027

**Published:** 2023-12-20

**Authors:** Kari Pahlman, Jane Williams, Diego S Silva, Louis Taffs, Bridget Haire

**Affiliations:** Sydney Health Ethics, School of Public Health, University of Sydney, Australia; ACHEEV, School of Health and Society, University of Wollongong, Australia; Sydney Health Ethics, School of Public Health, University of Sydney, Australia; Sydney Health Ethics, School of Public Health, University of Sydney, Australia; Sydney Health Ethics, School of Public Health, University of Sydney, Australia; Kirby Institute, University of New South Wales, Australia; Australian Human Rights Institute, University of New South Wales, Australia

## Abstract

In late March 2020 in response to the COVID-19 pandemic, Australia introduced mandatory 14-day supervised quarantine at hotels and other designated facilities for all international arrivals. From July 2020, most states and territories introduced a fixed charge for quarantine of up to $3220 per adult. The introduction of the fee was rationalised on the basis that Australians had been allowed sufficient time to return and there was a need to recover some of the cost associated with administering the program. Drawing on an empirical study of 58 returned Australian citizens and residents quarantined between March 2020 and January 2021, this paper aims to explore how people experienced paying for hotel quarantine, particularly with respect to fairness and relatedly, the principle of reciprocity. Reciprocity requires that the state has an obligation to assist individuals in discharging their duty to comply with public health measures and avoid disproportionate burdens accruing to populations or individuals. Though participants had varying opinions on whether they thought it fair to be charged for their quarantine, for many, the fee constituted a significant burden and source of stress. Given the undertaking of quarantine is primarily for the benefit of the public good, we argue the financial cost imposed on individuals does not meet the demands of reciprocity. It is imperative that future quarantine and isolation arrangements consider seriously the need to minimise burdens of individuals subject to such measures, and that fees do not become a new norm in public health and infectious disease control.

## Teaser

In March 2020 in response to the COVID-19 pandemic, Australia introduced mandatory 14-day supervised quarantine at hotels and other designated facilities for all international arrivals. From July 2020, most states and territories introduced a fixed charge for quarantine of up to $3220 per adult. The introduction of the fee was rationalised on the basis that Australians had then been allowed sufficient time to return, and there was a need to recover some of the cost associated with administering the program. Drawing on an empirical study of 58 returned Australian citizens and residents quarantined between March 2020 and January 2021, this paper aims to explore how people experienced paying for hotel quarantine, particularly with respect to fairness and relatedly, the principle of reciprocity. Though participants had varying opinions on whether they thought it fair to be charged for their quarantine, for many, the fee constituted a significant burden and source of stress. Given the undertaking of quarantine is primarily for the benefit of the public good, we argue the financial cost imposed on individuals does not meet the ethical demands of reciprocity.

## Introduction

As part of an early COVID-19 suppression strategy, Australia’s federal, state and territory governments introduced a suite of border control measures, including mandatory 14-day supervised quarantine for international arrivals at designated hotels or accommodation facilities (hereafter, mandatory hotel quarantine). This policy was announced by the Australian Federal Government on 27 March 2020 and came into effect less than 48 h later, set up and administered by states and territories (Australian Parliament House, 2020). While the federal government has oversight of the international border, individual states and territories were responsible for implementing quarantine for arrivals into their jurisdiction. Individual quarantine facilities were responsible for managing the day-to-day implementation of quarantine rules. While many states used hotels, one jurisdiction, the Northern Territory, used a repurposed mining camp in place of hotels for quarantine. These varied management arrangements across the country meant that there were differences in how quarantine was executed and experienced depending on the state of arrival and the facility to which travellers were allocated. In the first 18 months of the program, almost all of the 452,550 people who arrived internationally by air went through the quarantine system (Halton, 2021).

Initially, mandatory hotel quarantine was funded entirely by state and territory governments, however, most jurisdictions introduced a fixed charge in July 2020, moving toward a partial user-pays model (Halton, 2021). The fee varied slightly depending on jurisdiction and the number of travellers quarantining together, generally around $3000 per adult with lower fees for additional adults and children sharing a room. Fees were fixed regardless of the designated facility; however, hotels also had the discretion to offer better food and accommodation options for additional premiums. Some hotels charged up to an additional $100 per night for rooms with balconies, for example (Tobin, 2021).

One justification for imposing fees in mid-2020 was that Australian citizens and residents at that time had been allowed sufficient time to return, and there was a need to retrieve some of the cost associated with administering the program (NSW Government, 2020). Some jurisdictions, such as New South Wales (NSW) and Western Australia for example, stated the fee was a contribution to reduce ‘the financial burden of COVID-19 on … taxpayers’ (see Department of Health, 2020; Revenue NSW, 2021). The fee in some states was also presented as being calculated based on a daily room and meal allowance (see Department of Health, 2020; Queensland Government, 2023). All states and territories had mechanisms by which people could apply for payment plans and fee waivers on hardship grounds; however, generally this application could only be made after an invoice was received which usually were issued at some point after quarantine was completed (see Coronavirus Victoria, 2021; Revenue NSW, 2021; Queensland Government, 2023). This meant that at the time of travel and quarantine, returning residents did not know whether hardship applications would be successful in reducing the financial costs of their stay.

While quarantine is a common public health intervention employed by states to limit the spread of infectious diseases, the restrictive nature of such a measure means its use requires clear justification. Often, discussions on the permissibility of quarantine have focussed on the conditions or principles required to satisfy infringement of liberty, including, for example, the harm principle, the principle of the least restrictive alterative, and the principle of proportionality (Upshur, 2003; Cameron *et al*., 2021). Reciprocity is another key principle in public health ethics literature used as a condition that must be met to justify restrictive measures (Upshur, 2003). Based on notions of fair play and mutual regard, reciprocity, according to Viens *et al*. (2009), demands that the state has an obligation to assist individuals in discharging the duty of those individuals to comply with public health measures where warranted, and to minimise associated burdens. Where such measures are restrictive and where individuals are disproportionately burdened or subject to unfair treatment, they ought to be appropriately compensated by the state (Viens *et al*., 2009). Building on this conception, Silva *et al*. (2016) define reciprocity as the obligations that arise when a person is burdened as a result of acting toward the public good, even if they also derive benefit. Such obligations, for example, in the form of compensation or restitution, must be commensurate to the burdens involved (Silva *et al*., 2016).

Indeed, while beyond the scope of this paper, reciprocity has been interpreted as the fundamental way of promoting fairness, or even as the virtuous disposition to be fair (Becker, 1986). Reciprocity as fairness has been discussed in the wider context of the COVID-19 pandemic, for example, in relation to what is owed to healthcare workers and others who take on exceptional risks by continuing to work for the common good, or what is owed by those burdened by the collective response to the pandemic more broadly (Fenton, 2021; Randall and Bernstein, 2021).

With respect to quarantine specifically, reciprocity has previously been discussed primarily in terms of both providing the conditions and support required for people to be able to discharge their duty (to the common good) to quarantine, and minimising associated harms and burdens to the greatest extent possible (Silva and Smith, 2015). This concerns not only providing sufficient and appropriate accommodation, food, medical and psychological support, but also ensuring that individuals are fairly compensated where they incur losses (Upshur, 2003). Existing literature related to reciprocity and quarantine generally prescribes that where compliance with such an intervention for the public good carries significant personal financial cost, these costs should also be compensated (Gostin *et al*., 2003; Holm, 2020).

Drawing on this principle of reciprocity, one could, therefore, reasonably conclude that Australian governments had a responsibility to first, provide the conditions reasonable to support individuals in quarantine, and second, to ensure those individuals were not unfairly burdened in doing so, including financially. Drawing on an empirical qualitative study with Australian citizens and residents who went through mandatory hotel quarantine, this paper aims to further explore some of the ethical considerations of hotel quarantine with respect to the principle of reciprocity, including as it relates to the requirement of individuals to pay for mandatory hotel quarantine. We argue that where undertaking quarantine is directed primarily toward the public good, the financial cost imposed on individuals does not meet the demands of reciprocity.

## Methods

This paper is guided by empirical bioethics methodologies that emphasise the relationship between theory and practice (Carter, 2019). Empirical bioethics is highly applied; it situates ethical analysis in a well explored and understood context. We used qualitative methods to develop a deep understanding of a particular experience and normative analysis to consider the place of reciprocity in mandatory supervised quarantine.

For this paper, we adopted a reflexive thematic analysis approach, a widely used method in qualitative research (Braun and Clarke, 2017, 2019). Reflexive thematic analysis is an approach that is both rigorous and systematic and also fluid and recursive, as patterns of meaning are identified and interpreted through a process of coding and theme development (Braun and Clarke, 2006). It aims not to simply summarise the breadth of the data, but interpret key features as guided by a research question that itself can evolve throughout the analysis (Braun and Clarke, 2017). The initial research question that guided our thematic analysis was broad: how did people experience having to pay for hotel quarantine? Coding was conducted inductively by KP, with themes generated and refined through frequent discussions with the research team, including JW and BH who conducted the initial interviews and prior analyses. This process of collaboration is itself central to the approach of reflective thematic analysis, as researcher subjectivity and reflexivity is understood as a resource to knowledge production (Braun and Clarke, 2019).

This paper draws on a subset of interviews with returned Australian citizens and residents, aged between 19 and 75 years, who had been quarantined in a designated facility between March 2020 and January 2021. Some had travelled for family or work-related reasons, or were returning from holiday, while others were moving home having been living abroad. Participants were recruited via social media (primarily Facebook groups related to hotel quarantine in Australia) and word of mouth, and were compensated for their time. Interviews were conducted by zoom or telephone by JW and BH; they lasted between 35 and 95 min with a median of 51 min using an agreed flexible question route that was designed to be responsive to participants’ circumstances (see Supplementary Appendix). Interviews took place over three distinct temporal phases that corresponded to a different quarantine policy. Phase 1 (March to May 2020), *n* = 30: 15 who had completed 14-day quarantine at home before hotel quarantine was mandated (not included in this analysis), and 15 people who had experienced mandatory hotel quarantine in its early days (March 28 to May 2020). Phase 2 (April to June 2020), *n* = 11: participants who had quarantined in two hotels in Victoria which were identified as the source of a major 2020 outbreak in Melbourne which contributed to the cessation of hotel quarantine in that state. Phase 3 (August 2020 to January 2021), *n* = 32: participants who were required to pay for mandatory hotel quarantine. The dates of the phases indicate the dates participants quarantined rather than the date of interview. Where quotes from participants are used, the state and date refer to the quarantine location and time. All participants are referred to by pseudonyms.

This study was highly collaborative and Phase 1 interviews took place while the interviewers were themselves under ‘Stay At Home’ COVID-19 orders. They reconvened after each interview to discuss what had been said, what was new, and also to debrief given the general difficulty and uncertainty faced by participants and, to a lesser extent, the researchers. Some interviews were harrowing, others were very straightforward. The interviewers managed this tension, and the unpredictability associated with each interview, by regularly checking that the flexible interview route was fit for purpose. The analysis phase was iterative and involved checking ideas against the breath of transcripts to ensure that it was not simply the most colourful or emotionally charged experiences that shaped researchers’ understandings of context. The authors are committed to justice in public health policy and action; we came to this paper with the assumption that reciprocity has a role where people are asked to take on certain types of burden in the name of public health for the benefit of others.

Ethics approval for this study was granted by the University of New South Wales (HC200275).

## Results

This paper draws on interviews with 58 participants—19 men and 39 women. Of these, 32 participants (Phase 3) quarantined after the introduction of fees, while 26 (half of Phase 1 plus Phase 2) did not have to pay for quarantine. We included their perspectives in this analysis because it was common for them to raise payment as a hypothetical, unprompted, when they were talking about their quarantine experiences. The bulk of the analysis, however, is drawn from Phase 3 participants who were asked about their thoughts about paying for quarantine, following the addition of a specific question to the interview guide. Of the 58 participants, 52 were quarantined in hotels, with six Phase 3 participants quarantined at the repurposed mining facility Howard Springs in the Northern Territory.

We report on two key aspects related to participants’ experiences paying for their mandatory hotel quarantine stay. First, the way the fee increased the stress for many participants, both in terms of the cost itself and the uncertainty related to the process of applying for waivers or payment plans. Second, and relatedly, we report on people’s perceptions of fairness in this context. Although participants did not explicitly use the term ‘reciprocity’, they did often discuss in terms of ‘fair’ or ‘fairness’ in relation to being charged for quarantine. As noted previously, reciprocity is related to notions of fair play and fairness; we draw the connection between these concepts and discuss findings in relation to the principle of reciprocity again later in the discussion.

### Payment as a Burden or Source of Stress

The cost of hotel quarantine, for many, was reported as a source of financial stress. The fees also, for some, compounded other COVID-19-related financial stresses. Several participants, for example, stated that returning to Australia meant leaving jobs abroad and that the cost of quarantine was particularly stressful while unemployed and facing significant uncertainty about future prospects.

*I had to resign my job in [country] in order to move here, without any employment security, and I’ve only had like certain amount of savings for me to last. And given the economy with the pandemic, it’s just going to be harder for me to get a job. Knowing that, plus knowing I might have to pay for the quarantine fee, it’s stressful just to think about it.* (Charlie, QLD, January 2021)

Some indicated they would also need to rely on family members, payment plans, or debt to pay for the quarantine bill, if a waiver was not granted.

*Obviously I’ll just have to pay that off [quarantine bill]. But the thing is, it’s just accumulating debt. Like I got made redundant last year at my work … so just having this extra bill. I just hope they don’t pressure us to pay it [quickly].* (Toby, NT, January 2021)

*I had a $3,000 quarantine bill … they gave me one month to pay it. I rang them when I got here, and I said ‘look, because I’ve used my credit cards to pay for the $26,000 [in flights], I’m completely maxed out, I need to get paid before I can pay this’.* (Martina, NSW, January 2021)

Indeed, as was the case for Martina, above, some participants expressed frustration that the contribution fee was additional to what was an already expensive exercise in returning home, particularly given the increased cost of air travel at the time due to border closures and government-imposed caps on arrivals. Seats on flights were scarce and many participants reported purchasing business class fares in order to secure passage, often having borrowed money or used savings to do so.

*We spent so much money on our flights to get home … it’s our house deposit basically.* (Kay, NSW, December 2020)

There were also additional and separate costs of undertaking quarantine for many, including having to purchase goods and services when it was felt that what was provided was insufficient or inappropriate. The need to purchase food in particular was mentioned a number of times. Tara, for example, expressed concern about the cost of supplementing food where her medical dietary requirements were not met:

*The food was such an issue. I am a celiac so I am quite severe, I get really bad migraines and rashes if I eat gluten and the hotel just could not get their head around that. They were giving me a meal made of barley and I would explain to them it’s not gluten-free, and they would say, ‘Yes, it is’. And it’s like, it’s not. And then I’d eat it, trusting them, and I’d get sick. … Thankfully we were allowed to order UberEats but, again, because it’s not cheap to come home during all of this, it’s just too much to be paying fifty bucks a meal three times a day.* (Tara, QLD, April 2020)

*So … probably every second or third night I’d order UberEats for dinner for the both of us … ended up spending probably close to $1,000 in food because what was supplied was just inadequate.* (Lloyd, NSW, June 2020)

The cost of supplementing food for children in particular was raised several times by parents who remarked that the meals provided for children were often inappropriate, or not delivered at suitable times. As explained by Sylvie:

*We had to buy it ourselves. We spent over $100 because the food was actually not suitable for a sixteen-month-old child. It said ‘toddler’ on it, but it was just a smaller version of an adult meal … there were some meals that were just completely unsuitable for children … Or other things where, for example, we were getting served at around seven-thirty, eight pm. Our child’s bedtime is seven-thirty, and the food was arriving seven-thirty.* (Sylvie, NT, January 2021)

Where participants needed to purchase goods that were not supplied in quarantine, the minimum spend for delivery as required by supermarkets and other stores—usually $50—also increased costs. Several expressed frustration at being in a situation of not only having to purchase things that should have been supplied, but also things they did not need to reach the minimum spend in order to get what they did need.

In addition to food, a few participants also noted the need to pay for supplies needed to wash clothes and clean rooms. One participant even reported purchasing a pillow to be delivered, as she felt that her room was not sufficiently clean or liveable.

*The cleanliness … In our room we had stains on the chairs and all that, look, I can look past some of that stuff. I can tell you when you see a pillow that’s all yellow I would rather not know. I actually couldn’t sleep on the pillow. We actually ordered a pillow in.* (Terri, VIC, April 2020)

In this sense, those in mandatory hotel quarantine already had non-trivial financial costs associated, including to purchase goods that should otherwise have been reasonably provided and the fee constituted an additional stress and burden. This was, for some, also amplified by the uncertainty in applying for a waiver or payment plan. Several noted that they were not able to apply for exemptions until after completing their entire quarantine stay and receiving the invoice. As such, they did not have a full idea of the true cost when making the decision to return home and undertake mandatory hotel quarantine. The lack of information about the payment process, and the importance of such in reducing this source of stress, was also identified from the interviews.

*We haven’t been given any information about, like we’re in a position where if we have to pay upon release, we can pay, but we have been given no information. Like nothing when we check in, none of the papers when we checked in said what the payment. If we were stressed about that, there definitely could be more communication and information in that regard.* (Caroline and Russell, NSW, December 2020)

The lack of information about the quarantine process more broadly was identified by participants as a source of significant stress. Indeed, the financial pressures reported here are in addition to previously reported non-financial costs and burdens of undertaking quarantine, including with respect to feelings of uncertainty and diminished agency, as well as exposure to risk (Haire *et al*., 2022; Williams *et al*., 2022).

Ultimately, the interviews show that the introduction of fees was a key source of stress and financial burden for some returning citizens and residents. For many, it was also on top of and additional to other costs related to returning to Australia during the pandemic and undertaking quarantine.

### Was It Fair?

Setting aside whether they were placed under financial stress, many participants discussed the contribution fee more generally in terms of fairness and whether it was reasonable they be charged for mandatory hotel quarantine. As noted earlier, while participants were not asked explicitly about the idea of reciprocity, several participants offered their perspectives about fairness in this context. Participants were broadly supportive of the requirement that people quarantine and many acknowledged the cost of the program. Some also expressed they did not think the cost should necessarily fall on taxpayers, particularly for non-essential travel. However, there were varied thoughts about whether it was fair that individuals undertaking quarantine themselves be required to bear that cost. A couple of participants raised the point that quarantine is largely for the benefit of the public and questioned whether it was therefore fair to ask the individual who is providing that benefit to pay the cost of such. Others similarly questioned whether it was fair or reasonable for the government to charge for something they understood to be a government responsibility.

*Well, somebody else made the point, which I thought was interesting, that, that the reason that you’re quarantining is to benefit the public rather than to benefit yourself. And so that argument was that it was unfair for them to have to pay for something that was ultimately for the benefit of others I guess.* (Renee, NT, January 2021)

*I’ve read that the government shouldn’t be charging you because it’s their responsibility, because it’s like a health and safety thing … Prisoners don’t pay.* (Nina, NSW, January 2021)

*.… deciding how much the user pays, how much the Government funded, it seems like an awful lot … But then I’ve read it costs up to eight thousand per person to provide the whole service.* ^1^ *And I certainly think the Government should pay quite a bit of that because it’s really protecting the people of Australia.* (Eloise, QLD, January 2021)

Indeed, these participants (indirectly) drew on this idea of quarantine as being for the public benefit and thus questioned to some extent whether it is therefore fair that they as individuals be required to pay for it. This notion of quarantine as being for the collective good is something that is central to the principle of reciprocity, as highlighted in the Introduction.

Some participants also expressed concerns that all returned travellers faced the same quarantine fees irrespective of individual circumstances and suggested fairer (their term) ways for payments to be managed so that people would not be unreasonably burdened or prevented from returning to Australia. Alternative suggestions included means testing and a tiered accommodation system where fees reflected the quality of service provided and included low-cost options.

*I do think it’s not fair [paying]. I think they should be provided with it because you’ve got people … even if it was means tested or something because you’ve got people, families, who just, there’s no way, that’d just break the budget.* (Ainsley, VIC, April 2020, quarantined prior to the introduction of fees)

*I mean I know other people who have not come home because of that cost [of quarantine].* (Diana, NSW, September 2020)

Considerations about the fairness of paying for mandatory hotel quarantine were also often contextualised in terms of the purpose and timing of travel. Several participants returning to Australia from lives in other parts of the world thought it was not fair to be charged, because they were not ‘just holiday-goers’. One participant also drew the distinction between those who were returning home, once, for good, for whom quarantine should be provided free, and those who were coming and going multiple times, including on business. Another suggested that being charged for mandatory hotel quarantine was also more justifiable in certain circumstances, such as for people who left Australia after the borders closed. As Ben stated:

*I don’t think it’s unreasonable that I pay, especially in my situation because I was already in Australia [and] I chose to travel overseas and then to come back. I do feel for some of the people that sort of got stuck overseas and have been trying to get back to then get back and get hit with a $3000 bill. I think that could’ve been better managed.* (Ben, SA, January 2021)

Other participants, however, reiterated that everyone had different circumstances and reasons for travelling when they did, including after the closures of borders due to unforeseen circumstances such as family illness and death. Moreover, several participants indicated that the introduction of the contribution fee on the basis of having already had time to return home earlier also did not reflect the reality of why and when people were able to travel home. Some reported practical constraints, such as having to work out notice on jobs, finish the school year, or expensive leases. Others had delayed travel in order to reduce risk of infection, or had faced lengthy logistical challenges getting flights.

*The … things that I objected to is you know … the cost. I do understand that it is costly … if a government has to pay for it, but you know, I didn’t really think that it was that fair because I was trying to come home before they started charging people. And you know, I was told a) by the Australian consulate if I was safe to just sit and wait and b) I did have original flights beforehand, but you know all those cancellations …* (Margarita, QLD, October 2020)

Indeed, the challenges with international transit was highlighted by several participants and the contrast between quarantine arrangements for domestic and international travel in this context was also made.

*So a lot of people did what we did where they were overseas and they were safe where they were. So they thought ‘we won’t rush back with billions of people rushing back, where we’re exposing ourselves, we’ll wait until it peters out and then we’ll go home’.* (Isobel, VIC, June 2020)

*It is offensive that someone who decided to go to New South Wales for Christmas to see their family and didn’t come back in time doesn’t get charged, but someone who [was overseas did] … It’s this perspective ‘well if you were overseas you should’ve come home’ [but] if I had’ve come home then, I would’ve been a burden on the system in other ways. I wouldn’t have been denied any welfare because I was in Australia. I wouldn’t have been denied any medical care because I was in Australia, and I’m an Australian citizen. But there’s this idea of people who’ve come back later have somehow given up those rights which isn’t fair. The other thing that I think grates me is the wording. I know that the word ‘traveller’ is literally what we’re doing … but I’m not travelling back from the U.S. because I’ve been on holidays, I’ve been living there for five years.* (Jade, NSW, January 2021)

There was frustration among some at the positioning of the policy as needing to reduce the burden on the taxpayer. A number of participants pointed out that those overseas were not costing the taxpayer in ways they would have if they had been in Australia, such as through eligibility for COVID-19 relief payments or medical care.

*[L]ike the government was giving out money in July, August, October, whenever. And if you’re overseas you don’t, you weren’t entitled. Like you know, there were families, there were people over that were going over for two weeks, or they were going over for a funeral, and they got stuck, and they got nothing offered because … it was like ‘you chose to go over’.* (Toby, NT, January 2021)

*[N]obody disagrees with quarantine, but I’m a taxpayer already. There’s an atmosphere out there that we’re getting it free and that I’m not a taxpayer contributing. I’m still paying tax, I’m still working in an Australian place. And, even if I wasn’t, my citizenship should afford me some protection because I don’t have another citizenship, you know.* (Renee, NT, January 2021)

This frustration extended to the lack of assistance provided by the government for returning home, as well as a perceived narrative at the time that returning citizens and residents were not welcome. The charge for mandatory hotel quarantine was viewed by some as an extension of the abandonment they had felt.

*There’s people that just don’t understand how little the government is actually assisting people in returning. They do almost zero and you’ve got quite a hard battle yourself. So you’re coming back feeling like you’ve had to battle your own country. And then you’re faced with a $4,000 bill … It was fairly costly experience to get back with zero help, and so you have that accumulation of feelings around no help, plus lots of money, plus a community that was quite happy for it to be that way making you feel that the community isn’t supporting you.* (Joseph, NSW, January 2021)

Findings show that many participants not only expressed that the financial cost of quarantine was a source of stress, but also questioned whether or not the fee and being placed under such stress was ‘fair’. Such questions and queries around fairness included whether quarantine is necessary for the collective good, as well as the use of blanket charges that did not take into account personal financial circumstances, or reasons for travel, for example.

## Discussion

While participants generally agreed with the need for mandatory supervised quarantine to minimise the spread of COVID-19 in Australia, our study shows that paying for quarantine constituted a burden for returning citizens and residents. This is in addition to the already significant risks and burdens that those returning took on by undergoing mandatory hotel quarantine (Dinh *et al*., 2021; Haire *et al*., 2022; Williams *et al*., 2022; Fotheringham *et al*., 2023). It also showed that many participants felt such a burden was not necessarily fair.

### What Does This Mean for Reciprocity?

As indicated in the Introduction, the concept of fairness—one frequently raised by participants including with respect to the financial cost of quarantine—is closely connected to the principle of reciprocity. Reciprocity is based on notions of fair play; it aims to promote fairness. Writing on reciprocity in public health, Viens (2008) states that the concept of reciprocity is that ‘one should return the good that one has obtained in an appropriate amount or should offset the harms that others have incurred performing acts on your behalf or acts from which you directly benefit (p. 1)’. Indeed, the idea that each party in mandatory quarantine (the quarantiner, the quarantined) owed something to the other was frequently raised in our study, often in the context of fairness.

Within the ethics of quarantine literature, and of public health interventions broadly, reciprocity is increasingly invoked as a key principle upon which such measures can be justified. Existing literature in this context generally holds that those who are subject to mandatory quarantine for the benefit of the public should therefore be supported to minimise to the extent possible the impact of such a burden, including, for example, to minimise or potentially compensate financial costs incurred (Upshur, 2003; Viens, 2008; Silva and Smith, 2015). Holm (2020), for example, in exploring compensation in the context of public health interventions such as quarantine, employs the principle of reciprocity to highlight that where a person has ‘freely provided an important good for society … letting them carry the full costs themselves would not be a fitting and proportionate response (p. 41)’. Meanwhile, Gostin *et al*. (2003, p. 3234) argue that fairness and equity would require that where people are obligated ‘to forgo their freedom for the common good (p. 3234)’, the financial burden of such should not be borne by the individual alone but the common as a whole. The authors discuss the examples of providing those undertaking quarantine with cash compensation, or ensuring sick pay or basic welfare benefits be provided (Gostin *et al*., 2003, p. 3234). Various state and territory public health legislation in Australia do have compensation clauses, including for where a person suffers ‘loss or damage’ due to being detained, that is, quarantined or placed in isolation. In some jurisdictions, however, these clauses state that compensation is not entitled as it relates to the COVID-19 emergency specifically (see ACT Health, 2023; Queensland Health, 2023). We will return to this idea of COVID-19 exceptionalism and the risks for reciprocity shortly.

According to the principle of reciprocity, those undertaking mandatory hotel quarantine for the primary benefit of the broader Australian public ought to not to be materially worse off in doing so. While the question of compensation for lost income or other burdens resulting from mandated quarantine is one that we set aside for now, we argue that at minimum, state and federal governments had an obligation to not *increase* burdens. The introduction of contribution fees, as demonstrated by the findings of this study, constitutes such, increasing the burden for many people in discharging their public health obligations. As highlighted in the results, the cost of quarantine was indeed a significant source of financial stress. For some participants, this burden was also on top of already not insignificant financial and non-financial costs and losses occurred during the 14-day period; many returning home were unemployed, had exhausted savings, were relying on financial help from family members, or going into debt to pay the cost of quarantine. Fees for mandatory hotel quarantine thus adversely shifted the balance of benefits and burdens necessary to obtain the public good, creating additional stressors and burdens not otherwise compensated for in other ways.

### Reciprocity and COVID-19 Exceptionalism

Australia appears to have breached its legal duties as stipulated under the International Health Regulations ((IHR, (2005)), which it freely signed. Indeed, reciprocity is embedded within the IHR (2005), which is currently the primary international legal instrument intended to regulate the public health response to the international spread of disease. Article 40 of the IHR (2005) expressly prohibits charging individuals for quarantine, specifying no (financial) charge shall be made by a state for quarantine requirements where such measures are ‘for the protection of public health’ (World Health Organization, 2005). The WHO Health Emergencies Programme Executive Director reiterated this in 2021, stating that an underpinning principle of the IHR (2005), including in the context of hotel quarantine, is that travellers ‘should not suffer economically from measures that are used to improve and protect the health of the population as a whole because that then is unfairly burdening an individual with the costs of a public health action (World Health Organization, 2021)’. Again, that those undertaking quarantine in Australia—for the benefit of the public good—suffered economically is something that was demonstrated in the findings of this study, as indicated in the first theme of the results.

We acknowledge the current debate about the IHR (2005)’s usefulness in managing the challenges of the COVID-19 pandemic, a debate underpinned by widespread non-compliance with the instrument (Ferhani and Rushton, 2020; Lee *et al*., 2020; von Tigerstrom and Wilson, 2020). We also note, however, that the IHR (2005) are legally binding on the 194 member states, including Australia. Yet many of the restrictions and exclusions imposed during the pandemic not only in Australia but across the world, have been justified by governments through an appeal to exceptionality and crisis (Foster *et al*., 2021). The framing of the pandemic as exceptional in this way, and the invocation of extraordinary powers thus enabled the largely unconstrained enactment of restrictive measures—even if temporarily—that would otherwise have been unacceptable, and many of which were inconsistent with existing legal standards (Foster *et al*., 2021; Bennett *et al*., 2022). The exclusion of COVID-19-related detention from compensation clauses within the Public Health legislation of some Australian states and territories is an example of this exceptionalism.

Certainly, the COVID-19 pandemic has demonstrated the need for emergency powers to respond effectively and quickly to infectious disease outbreaks and other public health emergencies. In Australia, emergency powers are legislated to enable expedited and larger-scale responses where required. However, such powers should not be used without careful checks and balances, and the at times disproportionate use of these powers during the COVID-19 pandemic—including with respect to border closures—continue to be discussed widely (Simic and Rubenstein, 2023). There are also risks of this kind of exceptionalism; appeals to the crisis as being unprecedented and thus justifying an exceptional response may set a new precedent, undermining accepted public health principles and relevant protections. With respect to quarantine, this has potentially important implications not only for any future pandemic, but also existing protocols for other infectious diseases. In the case of tuberculosis (TB), for example, the notion of paying for quarantine or isolation would be particularly problematic, not least because as a ‘disease of poverty’, those disproportionately affected by TB are of lower socioeconomic status and who may not have the means to contribute financially (Silva *et al*., 2016). Just as importantly however, the transmission and distribution of TB burdens are, at least in part, due to the failings of governments to address the structural risk factors of TB and thus making individuals financially responsible for such would be morally troubling (Silva *et al*., 2016). It is imperative that the emergency powers used during COVID-19 in relation to quarantine and the requirement for individuals to pay for quarantine remain open to scrutiny so as not become a new (reciprocity denying) norm. As the data from our study demonstrates, the sidelining of reciprocity in the introduction of payment for participation in mandatory quarantine disproportionately increased the harms for those who may already be experiencing economic hardship.

### Quarantine Fees, and the Right to Return

Though reciprocity is not the only principle that ought to guide the implementation of liberty-restricting measures, the introduction of fees nevertheless makes the justification for mandatory hotel quarantine less tenable. Of course, one might object (as Australian governments have) that it would be unreasonable for the state to cover the cost of quarantine for all returning citizens and residents. The full cost of the mandatory hotel quarantine program to states and territories is to date unclear. However, the NSW government reported spending $65 million on quarantining international arrivals in the first half of 2020 prior to the introduction of the contribution fee in July 2020, while the Victorian government spent approximately $195 million by the end of September that year before introducing fees in December, for example (Coate, 2020; NSW Government, 2020). This is no small amount, and we acknowledge that any funds allocated to the hotel quarantine program were inevitably diverted from other public goods. However, the cost of the quarantine program was likely still less than the costs that would have been incurred had COVID-19 been circulating in an unvaccinated population. The justification of fees on a cost-saving basis is also complicated by the institution of other expensive programs during the pandemic such as JobKeeper (Borland and Hunt, 2023), which could be viewed as an act of reciprocity but for which many of those Australians forced to quarantine were not eligible and thus did not cost the state. This issue was raised by some participants, as highlighted in the results. In any case, as other scholars writing on reciprocity have argued, while cost is undoubtedly a relevant policy question, it does not minimise the duty of reciprocity or the moral—as distinct from practical—obligation of governments to minimise burdens on those disproportionately impacted by public health interventions (Holm, 2009; Silva *et al*., 2016).

Policy decisions related to quarantine fees can also be seen in the context of broader public and political discourses surrounding border closures and international travel. By the time fees were introduced in mid-2020, there appeared to be a general sentiment among the broader Australian population that those in quarantine were at fault for not returning earlier and thus were not owed any duty of reciprocity to minimise or compensate for the burdens they incurred. In Australia, there was a narrative directed toward those unable to return home, as summarised by Soutphommasane (2021), that ‘their failure to make it back [in time] must be counted as their just deserts, and not their misfortune’. The difficulties in returning and the general lack of support and assistance from the government toward the thousands of Australians stranded overseas—more than 45,000 still by October 2021—compounded these sentiments (Sheel *et al*., 2021). This feeling of abandonment was highlighted by several participants. However, what is also clear from our study is that the reasons people were travelling at different times throughout the pandemic were not always a matter of choice or frivolity; most participants were Australian citizens and residents returning home. At the least, in these particular circumstances, we maintain that where people are required to do quarantine for the protection of the wider public, they should, generally, not be required to pay for it.

In any case, it is not evident that these factors—purpose or timing of travel—are immediately relevant to the principle of reciprocity. It is difficult to justify the forgoing of a duty of reciprocity for something that people should be able to do at will, that is, return to their country of citizenship or residence (Silva, 2022). Indeed, the right to return is enshrined in international human rights law, including the Universal Declaration of Human Rights (UDHR, Article 13) and the International Covenant on Civil and Political Rights (ICCPR, Article 12), to which Australia is party. The requirement for people to pay for mandatory hotel quarantine arguably infringed upon this right to return by making it conditional upon payment of a fee high enough to be a barrier for many people (Jolkina, 2021; Pugh *et al*., 2021). As one participant indicated in the results, it is not difficult to imagine there are people who remained abroad because the cost of returning and undertaking mandatory hotel quarantine was prohibitive. We could also assume there would have been people who similarly could not leave Australia during this time (even if granted exception to do so) owing to the cost and difficulty of returning).

Certainly, one might object that even when there is no pandemic some people may not always be able to afford to return home from abroad and the right of return does not entail that a person has no costs in returning. However, a key difference with the cost of quarantine is that this is an extra charge imposed by the state in addition to the other ordinary logistical costs that a person may face in returning. Furthermore, in quarantining during a public health emergency, returning travellers are taking on burdens purely for the protection of others. Thus, they should be supported and/or compensated accordingly.

While the question of under what (if any) circumstances it is permissible to suspend the right of return—and the role of financial barriers in such—is outside the scope of this paper, we argue that the government still has an obligation to minimise the burdens on people in discharging their public health duties when returning home. Moreover, the duty of reciprocity holds irrespective of whether or not someone believes they are owed such duty.

### Limitations

The participants who volunteered to be interviewed for this study may have had particularly strong feeling about their experiences of quarantine, including being required to pay for it, and thus may be over-represented in the study group compared with the broader population that experienced mandatory hotel quarantine. We also recognise that the participants included are those that in one way or another were able to afford to return, noting not only the cost of quarantine but also of exorbitant airfares during a time when incoming flights were limited (Tobin, 2020; Visontay, 2020). However, we nevertheless consider it to be a large and rich dataset for a qualitative study, with 58 interviews conducted across 11 months, 5 states and territories, and a range of policy settings. There were many similarities in the stories and experiences shared.

## Conclusion

Reciprocity is a key ethical principle that liberty-restricting measures such as mandatory hotel quarantine must meet in order to be justifiable. According to such principle of reciprocity, governments have a responsibility to ensure that individuals are not unfairly or disproportionately burdened in discharging their public health duty to quarantine. Drawing on an empirical study exploring the experience of paying for hotel quarantine in Australia, we argue that the introduction of the contribution payment by state and territory governments for returning citizens and residents during the COVID-19 pandemic in Australia ran counter to the demands of reciprocity, eroding an accepted and pre-existing standard that people should not pay for their own detention during public health emergencies. We thus argue that in future, programs of mandatory quarantine ought to ensure that those affected by the policy are appropriately supported, and not disproportionately impacted, financially or otherwise. In implementing such arrangements, governments—both in Australia and beyond—ought to consider more seriously the burdens imposed on individuals when acting toward the public good, and pay greater attention to obligations that then arise.

## Supplementary Material

phad027_suppl_Supplementary_Material

## Footnotes

1 The cost of servicing one person in quarantine is reported to be between $5500 and $9000 per person in some jurisdictions (Halton, 2021).
