# Supplementary material for: Reciprocity, Fairness and the Financial Burden of Undertaking COVID-19 Hotel Quarantine in Australia
Source: Public Health Ethics. 2023 Dec 20;17(1-2):67–79. doi: 10.1093/phe/phad027 (PMC11245694; doi:10.1093/phe/phad027)
Supplement: phad027_suppl_Supplementary_Material [file phad027_suppl_supplementary_material.docx]

## Appendix A

**Self-isolation research questions**

1. How well do people comply with self-isolation directives?

2. What factors facilitate compliance or non-compliance with self-isolation directives?

3. How do people in self-isolation navigate emerging and pre-existing health issues?

4. How do people in self-isolation manage psychosocial and emotional challenges?

5. How do caring responsibilities impact on self- isolation?

**Inclusion criteria:** people who have been or are currently in mandated self-isolation in Australia in 2020

**Semi structured interview guide:**

Introduction, verbal consent process, selection of pseudonym, discussion of emotional supports available if needed.

1. Can you tell me about when you were first told to go into self-isolation? (prompts – who directed you to isolate, what instructions were you given, did you have any inkling that you were likely to have to go into isolation before you received the directive?)
2. What did you feel worried about when you received the directive? (Do you have caring responsibilities that were affected? Other responsibilities that concerned you? Was your income safe?)
3. Can you tell me a bit about your home (or other isolation) environment (prompts – who do you live with, how large is the space, what are the shared facilities, what is the internet connection like, do you have pets?)
4. Did you have anyone to help with you managing the isolation process (prompts – did you have someone buying you groceries, medicine, sanitary supplies, entertainment, helping with caring responsibilities)
5. Was it clear to you whether you were allowed to go outside at all? (Prompt - such as go for a walk or run – did all the directive make sense to you, seem clearly justified)
6. What was your impression of the security arrangements? (Did you feel safe? Did you observe use of PPE?
7. Can you tell me about the payment? (prompts: did you have to pay? How does this affect you? Can you tell me about any waiver system?
8. What was the hardest thing for you?
9. Was there anything that made you feel that you couldn’t handle staying in isolation anymore (prompts - what were the triggers for this?)
10. Did you become unwell while you were isolated? (prompts - what were your symptoms, how did you treat symptoms, was there a healthcare provider you could ask, did you need to go to hospital or worry that you might need to?)
11. Was there a point where you needed to break the isolation directive (prompt- something that you needed and couldn’t get? Did you feel like there would be no real harm is making a small break in the rules?)
12. Did you see or hear of anybody breaking the rule while you were in quarantine?
13. Was there anything that helped make the process more bearable for you? Do you have any suggestions about supports that could be put in place for others who are self-isolating?
